# Supplementary material for: Monitoring methaemoglobinaemia in birds using 5 μL of whole blood
Source: PLoS One. 2023 Mar 16;18(3):e0282820. doi: 10.1371/journal.pone.0282820 (PMC10019692; doi:10.1371/journal.pone.0282820)
Supplement: Attachment — Submitted filename: MHb in birds micro_PONE 2022.docx [file pone.0282820.s001.docx]

Monitoring methaemoglobinaemia in birds using 5 μL of whole blood

Clive A. Marks 1,2,* , Katherine Trought 2, Samantha Brown 2, Jane Arrow 2, Brian Hopkins 2

**Reviewer’s comments**

This is a well-conceived study that has achieved its objective, to find a method of determining the level of methaemoglobinaemia in the small volume of blood that can be taken from small birds. This is needed for toxicological studies of the effects of baiting with a methaemoglobin-inducing agent (PAPP) on non-target species, mainly birds. The new method demonstrates how a selection of commercially-available devices can be combined with bespoke equipment to create a method not otherwise available. The study is well-conducted, the data analysis is sound, the findings are clearly presented and the conclusions are justified.

**Figures**

There are very many figures that show similar data. Perhaps some could be placed in a Supplementary section. This could make the narrative flow more easily.

**Minor issues**

Line no.

18,	total Hb is not HbFe^2+^, but needs definition here – later called Hbt (line 203)

44	word order?

80-81	re-order words?

142-	birds were given increasing doses of PAPP until a clinical response. The actual doses used varied between species and should be indicated.

203-6	give N animals used for each correlation analysis

298-	in this discussion of sources of variation in MAI, has consideration been given to the possibility of further oxidation products of Hb, such a sulf-Hb?
