# Supplementary material for: Monitoring methaemoglobinaemia in birds using 5 μL of whole blood
Source: PLoS One. 2023 Mar 16;18(3):e0282820. doi: 10.1371/journal.pone.0282820 (PMC10019692; doi:10.1371/journal.pone.0282820)
Supplement: Attachment — Submitted filename: Reply to reviewers.docx [file pone.0282820.s002.docx]

Robert Chapman, Ph.D.
Academic Editor
PLOS ONE

**16^th^ January 2023**

**Dear Dr Chapman,**

**RE: PONE-D-22-31643. Monitoring methaemoglobinaemia in birds using 5 µL of whole blood**

**We thank the reviewers for their comments and inputs to the MS and take the opportunity to respond and modify the paper accordingly (our additions in bold in this ‘response to reviewers’).**

**Your sincerely,**

**Dr Clive A Marks**

5. Review Comments to the Author

**We thank the reviewer for the very helpful comments and suggestions and we have incorporated or addressed each as follows:**

**Reviewer #1**: This is a well-conceived study that has achieved its objective, that sought a method of determining the level of methaemoglobinaemia in the small volume of blood taken from birds. This is needed for toxicological studies of the effects of baiting with a methaemoglobin-inducing agent (PAPP) on non-target species, mainly birds. The new method demonstrates how a selection of commercially-available devices can be combined with bespoke equipment to create a method not otherwise available. The study is well-conducted, the data analysis is sound, the findings are clearly presented and the conclusions are justified.

Figures
There are very many figures that show similar data. Perhaps some could be placed in a Supplementary section. This could make the narrative flow more easily.

Minor issues
Line no.
18, total Hb is not HbFe2+, but needs definition here – later called Hbt (line 203)

**Changed to ‘HbFe^2+^’ or referred to as ‘haemoglobin (HbFe^2+^)’ in other places in the MS. The confusion arises as the CeraChek device claims to measure Hbt, but in reality excludes HbFe^3+^. We have also changed this in the figures and text.**

44 word order?

**Now:**

**“Sodium nitrite (NaNO_2_) is another MetHb-former registered for the control of brushtail possums in New Zealand (****9****) and feral pigs in Australia (****10****).”**

80-81 re-order words?

**“CO-Oximeters used for laboratory or point-of-care analysis to determine the concentration of different haemoglobin species typically require minimum volumes ranging from 50 µL (****20****) to 300 µL (****21****). While blood samples as small as 45 µL have been lysed, centrifuged and added to a phosphate buffer to permit MetHb analysis in the laboratory (****22****), several factors suggest that rapid analysis of avian blood at the point of collection should be preferred.”**

142- birds were given increasing doses of PAPP until a clinical response. The actual doses used varied between species and should be indicated.

**In this section (142) this refers to NaNO2 and we state:**

**“A dilution series of sodium nitrite (NaNO_2_) was created in PBS (10 mM phosphate, 150 mM sodium chloride) to produce a concentration gradient of 2.5, 5, 10, 15, 20, 40, 60 mmol L^-1^ with a corresponding control (PBS only). A total of 100 µL of each sodium nitrite dilution (or control) was added to 900 µL of blood and incubated for a further 20 minutes while data were taken after 10 minutes when and asymptote for MetHb values had occurred.**

**In the section 192 we add:**

**“The overall range of PAPP doses used varied between 0.6 and 855 mg kg^-1^ and the full range of doses used in each species is reported elsewhere (****27****).”**

203-6 give N animals used for each correlation analysis

**These data refer to the *in vitro* data with NaNO2 that do not use multiple animals, so we did not appended the n.**

298- in this discussion of sources of variation in MAI, has consideration been given to the possibility of further oxidation products of Hb, such a sulf-Hb?

**We now add:**

**“However, if sulphaemoglobin (SHb) and carboxyhaemoglobin (HbCO) are present within the blood sample, the determination of MetHb and HbO by absorbance spectroscopy can be confounded under certain circumstances. Combined, SHb and HbCO are normally present at only 1- 2% (****44****) and are unlikely to interfere substantially with the MAI unless SHb is formed in far greater concentrations due to the presence of specific xenobiotic compounds and the action of some bacteria (****45****). Practically, only in the presence of significant atmospheric concentrations of carbon monoxide gas will HbCO levels become elevated enough to interfere in the determination of HbO and influence the MAI.”**

**Reviewer #2:** The concept of measuring metHb concentration in small blood volumes has merit. However, this manuscript does not explain the data in a way that is useful and can be understood by others significantly familiar with the field. There are fundamental problems with the way the MAI is calculated in that the peaks at 575 nm will never go to zero and the contribution of the MetHb to the peak at 575 needs to be accounted for in all measurements. If the authors plan on submitting this manuscript again, it needs to be redone to improve the clarity of sentences.

**We have altered several sections to better explain aspect that may have been unclear that have been pointed to by other two reviewers. Reviewer 2’s main contention is suggesting that a failure to permit absorbance measurements at 575 nm to trend towards zero is a fundamental problem. However, we refute this. Firstly, we refer to the second paper on bird susceptibility to PAPP that has been accepted:**

**Marks CA, Trought K, Brown S, Arrow J, Hopkins B. Sensitivity of high conservation value birds to para-aminopropiophenone (PAPP) determined by sub-lethal dose-response assay. Animals. in press.**

**Here the MAI has been validated against bird survival data and this leaves little doubt that the MAI is highly informative irrespective of how we have used absorbance data at 575 nm. It can be significantly regressed with declining HbFe2+ until death. We offer the following detailed rebuttal to explain our reasoning:**

**For *in vivo* measurements predicting bird sensitivity, the 575nm absorbance peaks do not need to go to zero - and never will in living birds prior to their death. All lethal outcomes *in vivo* occur well before this point. As we have shown in the other paper, 99% of birds survived at a MAI of > 0.66 and 50% of birds are expected to die when the MAI is 0.53. This has been validated. Below an MAI of 0.5 lethal outcomes become progressively assured. There is little doubt that the MAI predicts survival in birds and fits a highly significant dose-response relationship. It does this because the MAI (absorbance @ 575 nm – absorbance @ 630 nm) quantifies the depth of methaemoglobinaemia in a single term and permits thresholds of hazard to be calculated. Alone, MetHb or HbO do not independently define the extent of a form of anaemia where one (MetHb) displaces the other (HbO). Accordingly, during methaemogolobinaemia MetHb and HbO are strongly inversely correlated – as we showed in our in vitro experiments. This was extremely useful as it acted as an “isosbestic-like” reference much more immune to error when determined in small blood samples that required well-controlled baselines. We tried many other data, approaches and data transformations, but overall, the simple MAI and its reciprocal proved to be the most robust and was key to the use of very small blood samples.**

**Determination of 575 nm peaks towards zero has relevance to measuring HbO *in vitro* (perhaps against a large gradient of NaNO2) but has no bearing on the *in vivo* MAI (absorbance at 575 nm [HbO] - absorbance at 630 nm [MetHb]. The MAI usefully predicts survival and lethal outcomes *in vivo* in birds dosed with PAPP - which was the primary objective of the study that five reviewers appear to have concurred with (2 in this PLoS paper and 3 in the *Animals* paper). In the PLoS paper, our preliminary *in vitro* data presented using NaNO2 validated the use of instruments and demonstrated relationship of HbO with MetHb and the viability of bird blood over time. We did not use the MAI in cases of in vitro analysis - as this would have been inappropriate. Neither did *in vitro* data with NaNO2 describe expected *in vivo* responses to oral PAPP (or any other MetHb-former), nor do they reflect PAPP/MetHb dose-response relationships that we observed and report in a second (*Animals*) paper. We speculate that the difference in measuring *in vitro* responses with NaNO2 and *in vivo* responses using PAPP, where the MAI has been used, may have caused some confusion. Irrespective, the criticism is erroneous as MAI values accurately predicted survival and mortality based upon threshold values in the same group of birds as we have used in both papers. The ability to measure a term (MAI) that predicts survival is what we set out to achieve, using very small blood volumes. Here, demonstrating the ability for 575 nm absorbance to trend to zero has no relevance, especially as it gets us no further towards being able to predict thresholds of survival and death that the MAI allowed.**

**That said, we are certainly theoretically able to produce an algorithm/relationship that models absorbance at 575 nm that trends towards zero and relate this to increasing MetHb. But to do so projects a trend that has no relevance to the observed *in vivo* response to PAPP. In contrast, the simple MAI requires no embellishment, is easily measured and has been validated in lethal dose trials as an index and marker of survival or mortality in birds. Regression of our MAI with declining HbFe2+ (using the CeraChek that is sensitive only to the presence of HbFe2+) was an independent method to demonstrate that the MIA generated *in vivo* was indeed strongly and linearly correlated with declines in Hb(Fe2+) until lethal outcomes were observed. In the second (accepted) paper we report that the MAI was highly successful in achieving this.**

**While we are quite open and realistic about the limitations and compromises involved in the approach we adopted, the suggestion that the data are flawed because of a failure to allow 575 nm absorbance to trend to zero is clearly not one of those limitations or compromises. That our approach has been validated against survival data in birds and HbFe2+ data *in vivo* is the main proof of the method. Problematically, no instrumentation (i.e. oximeters) existed to directly measure MetHb or HbO immediately *in vivo* in the small bird blood samples we needed to work with to do this work. Delays in analysis are certainly associated with changes in the very values (especially 575 nm HbO absorbance) that we needed to measure in close to real time. Hence, conventional lab approaches failed to allow us to collect valid sensitivity data in birds. Although we tried them, we could not use conventional lab methods to generate the comparative sensitivity data we required for small bird species. Our novel approach comes from a clear need, not a desire to ignore conventions or proven approaches, as there are none.**

**Reviewer #3**: This reviewer appreciates the care taken by the authors to develop methods that mitigate risks and death to study animals. Furthermore, obtaining point-of-collection data via spectroscopy on small sample volumes is very difficult, and this reviewer appreciates the achievements reported in the manuscript.

This reviewer recommends several modifications to the manuscript to clarify the presentation and interpretation of results prior to publication. Specific areas to be addressed are given below with line and figure references.

Line 34: The statement “…methaemoglobin (MetHb), it is rendered unable to release O2 from the tetrameric Hb molecule…” is misleading since MetHb is unable to bind and carry O2 (Mansouri and Lurie, 1993, American Journal of Hematology). This reviewer suggests modifying the text to clarify this point.

**We shared a similar understanding until recently reviewing the literature on this topic. Much to our surprise, it appears to be incorrect – but almost on a technicality. It is a really interesting dilemma within the literature itself. Here are the references that we have used below:**

**Wright RO, Lewander WJ, Woolf AD. Methemoglobinemia: etiology, pharmacology, and clinical management. Ann Emergency Med. 1999;34(5):646-56.**

**Jaffe E, Hultquist D. Cytochrome b5 reductase deficiency and enzymopenic hereditary methemoglobinemia. The metabolic and molecular bases of inherited disease 7th ed New York: McGraw-Hill. 1995:2267-80.**

**Bunn HF, Poyton RO. Oxygen sensing and molecular adaptation to hypoxia. Physiol Rev. 1996;76(3):839-85.**

**Mansouri A, Lurie AA. Methemoglobinemia. Am J Hematol. 1993;42(1):7-12.**

**We have revised our statement to:**

**“When oxidised to the ferric state (HbFe3+), known as methaemoglobin (MetHb), Hb is unable to bind to O2 as its sixth coordination site becomes occupied by a water molecule or hydroxyl group (1). Incomplete oxidation of any of the four Hb subunits (two α2 and two β2 chains) will impart an increased affinity for O2 that remains bound to one of the sub-units (2) causing a shift in the oxygen dissociation curve to the left and preventing its release (3).”**

**In effect, this does not change the practical reality; MetHb is unable to release O2 or take up further O2. Unfortunately, the above understanding also makes the statement that MetHb cannot ‘carry’ O2, technically incorrect. However, we would be the first to admit that finding the definitive data to support this is difficult for birds. We struggled with this for some time as most textbooks appear to repeat an oversimplification.**

Lines 136-138: It is unclear what volume of NaNO2 in PBS was added to blood samples to induce methemoglobin formation and whether the cells are lysed by the addition of this solution. Please add text to clarify and comment on whether cell lysis and subsequent reduction in light scattering has an impact on the spectroscopic analysis with the custom apparatus.

**Our protocol with the use of NaNO2 gradients followed the methods described by (****Shihana *et al.* 2010****) for human samples that was previously validated in dogs and cats by (****Patton *et al.* 2016****). These used an even higher concentration gradient of NaNO2 (> 60 mmol L-1) that we found caused hemolysis, so we restricted our comparisons to < 60 mmol L-1. This also alerted us to hemolysis as an outcome and we took some steps to monitor it.**

**Importantly however, none of these in vitro comparisons with NaNO2 used the customised device. This was used for two instruments (CeraChek and a CO-oximeter) to validate the inverse relationship of MetHb and the Cera-Chek determination of changing HbFe2+ (R2 > 0.98). It also showed that the strong relationship between HbO and MetHb (R2 > 0.96 in mammals) but failed in birds due to depletion of HbO by oxidative bird erythrocytes (shown in Figure 5). This was key data that led to the use of the MAI and the need to test bird blood immediately after collection. Although we did not use the higher NaNO2 concentrations used by other authors, neither did they report haemolysis as a confounding factor. We observed no incidence where NaNO2 and PBS caused lysis in preliminary trials with 60 mmol L-1 although this is something that we were alert to. At 414 nm and 490 nm absorbance we found no clear evidence suggestive of hemolysis. We also routinely inspected blood visually and microscopically and could find no indication that PBS/NaNO2 was causing lysis over the short duration where these data were generated (within 10 minutes). Preliminary trials with NaNO2 established 10 minutes as when all values asymptote. We certainly did detect signs of hemolysis, or suspect its presence in samples with far higher NaNO2 concentrations and after 24 hours especially.**

**In the methods we now clarify that:**

**“A dilution series of sodium nitrite (NaNO_2_) was created in PBS (10 mM phosphate, 150 mM sodium chloride) to produce a concentration gradient of 2.5, 5, 10, 15, 20, 40, 60 mmol L^-1^ with a corresponding control (PBS only). A total of 100 µL of each sodium nitrite dilution (or control) was added to 900 µL of blood and incubated for a further 20 minutes while data were taken after 10 minutes when and asymptote for MetHb values had occurred. ”**

**We have added the following text to clarify:**

**“We routinely attempted to assess presence of haemolysis by a combination of visual inspection against a white background, through light field microscopic examination and by comparing changes in absorbance at 414 nm (****30****).”**

**In the results we now say:**

**“After visual, microscope and spectroscopic examination at 414 nm, no indications of erythrocyte hemolysis were observed at any of the NaNO_2_ concentrations.”**

**After data in Fig 5:**

**“Visual and spectroscopic examination found no obvious hemolysis present.”**

**With reference to the MAI in the discussion we mention:**

**“Our preliminary trials sought to validate instruments against different MetHb concentrations produced with a NaNO_2_ gradient and followed the methods described by (Shihana et al. 2010) for human erythrocytes that were validated in dogs, cats and birds (Patton et al. 2016). We found that NaNO_2_ concentrations > 60 mmol L^-1^ caused varying degrees of hemolysis, so we restricted our comparisons to an upper limit of 60 mmol L^-1^.”**

**“Discrepancies between absorbance-based measurements of haemoglobins and those using biosensors reliant on different analytical principles may also occur due to significant haemolysis (47)…”**

Lines 184-186: It is unclear why the authors chose to report results in terms of a Methemoglobin Absorption Index (MAI) when the change in absorbance at 630nm (MetHb) should return the same information. The biggest concern with this is the convolution of the pure HbO spectrum with the pure MetHb spectrum, so while HbO is being depleted, MetHb is growing in at the chosen wavelength of 575nm. Will the authors please comment on this chosen reporting method and clarify in the text?

**Originally we tried to measure and use the independent absorbance (630 nm and 575 nm) in a very small blood sample, and associated them with MetHb and HbO and bird survival. Such independent measures of MetHb and HbO were far more error prone and we detail some of the reasons below. This led to the use of the MAI, especially as it was found to be a far better predictor of survival and thresholds of hazard in birds (as described in the now accepted Animals paper attached to the PLoS submission).**

**A range of issues (e.g. turbidity, possibly light scattering in the sample and different heme characteristics etc), required independent measurements to be referenced to isosbestic wavelengths. This is often done with a third (or additional) reference wavelength, but variations encountered between species made the use of independent measures (575 nm and 630 nm and a 3rd wavelength) more prone to error in some cases. We tried a range of isosbestic points yet the most stable metric used the proven association of MetHb with HbO. The MAI was validated in lethal dose trials in birds and thresholds based on the MAI found to be highly predictive of death and survival (where those based on MetHb and HbO alone were less robust). The MAI worked better than any other approach. Adding additional isosbestic points appeared to compound error in some species, possibly only because it was a third or forth term open to various measurement error and compound error and maybe because there is a small shift encountered for distantly related bird groups that had variable heme structure (e.g. kiwi v birds in the Neoaves). We suspect so. Overall, by using the MAI (absorbance @ 575 nm – absorbance @ 630 nm) we sought to quantify the depth of methaemoglobinaemia in a single term. Alone, MetHb or HbO do not independently define the extent of a form of anaemia where one (MetHb) displaces the other (HbO). Accordingly, during methaemogolobinaemia MetHb and HbO are strongly inversely correlated. This was extremely useful as it acted as an isosbestic-like reference much more immune to error when determined in small blood samples. We tried many data transformations, but overall, the simple MAI and its reciprocal proved to be the most robust in our bird survival trials that we report here:**

**Marks CA, Trought K, Brown S, Arrow J, Hopkins B. Sensitivity of high conservation value birds to para-aminopropiophenone (PAPP) determined by sub-lethal dose-response assay. Animals. in press.**

**We now include:**

**“Methaemoglobinaemia, is a functional anaemia where one haemoglobin (MetHb) displaces another (HbO), in a relationship that is strongly inversely correlated (****10****,** **27****). Individually, neither MetHb or HbO concentrations proved to be as successful at predicting bird survival as the MAI (****31****). In small blood samples, relative changes in absorbance between 575 nm and 630 nm appeared far less affected by error than those referenced to zero baselines that might shift slightly between samples due to imperfections in the cuvette repositioning or changes in the optical characteristics of the sample itself.”**

Line 187: It is concerning that there is a nonzero baseline for Figure 2. What is the origin of this and is the baseline consistent across all measurements? A nonzero baseline is typically an indication of scatter either from the sample or the sample holder. If scattering in the sample is dynamic and changing over the course of the experiment, this would affect the linearity of the MAI plot (Figure 2b). If it changes from one experiment to the next it could shift the MAI up or down with respect to the y-axis. This effect would be most pronounced in experiments where samples are subject to increased cell lysis due to higher ionic strength (increasing nitrite concentrations). This would impact the interpretation of data presented in figures 3 and 4.

**Figure 2 is a segment of the full absorbance spectra that was generated for each sample (300 – 1000 nm). The entire spectra was too detailed to publish without loss of resolution in the region we are concerned with. In this case a baseline was achieved in the near infra-red; where we were monitoring several other wavelengths (as well as UV for hemolysis). When these data were collected we were not certain what wavelengths would prove to be the most informative. The baseline has been consistently set, although there have been slight shifts observed. One of the main reasons we used the MAI was that relative changes between these two wavelengths (MAI = 575 – 630 nm) were used rather than referenced to the baseline. This greatly reduced error. One of the reasons why we used the index (MAI = 575 nm – 630 nm) was that while small shifts in the baseline due to sample turbidity (possibly due to lysis and sedimentation in the preliminary NaNO2 data) occurred from time to time, *relative* changes between the two wavelengths were far more immune to error than independent measures referenced to a third wavelength or baseline. This was also tested several times using blood samples and dyes and held true. Nonetheless, we agree that cell lysis can affect the quality of these data and we had no good measure of it incorporated in our approach. We think it unlikely to be a problem though with in vivo data, as we analysed samples of fresh blood within 1 minute and the high R2 and strongly significant fit between PAPP dose and the MAI, as reported in the other accepted paper (*Animals*), suggests that this is a relationship with good predictive power.**

**We’ve now better explain the calibration:**

**“For all *in vivo* bird trials, we used the Flame^®^ absorbance spectrometer with a sensor bandwidth of 350 – 1000 nm (Ocean Optics: Largo, USA) with a HL-2000 Vis-NIR spectrometer light source (Ocean Optics: Largo, USA) that has a spectral range of 360 - 2400 nm set up and calibrated using the manufacturer’s instructions for dark-field and light-field calibration with blank cuvettes. Absorbance data were generated over the full range of the spectrometer (350 – 1000 nm) from 3 µL blood samples pipetted into a disposable cuvette normally used by the DiaSpect^®^ Tm Hb analyser (EKF Diagnostic: Cardiff, United Kingdom).”**

**Our equipment was dark-field and light-field calibrated in each session with blank cuvettes – as a standard method for this Ocean Optics spectrometer. This was done before each lab session or when anything was moved or if settings were changed. Hence, the baseline was consistent. We monitored a unique near IR signal and also needed to monitor UV wavelengths for signs of haemolysis, this also made this off-set baseline setting necessary. Light scatter due to the optical coupling was minimised as far as possible; an extremely tight fit was developed using a 3D printer insert that received the 3 micro L cuvette that had a high manufacturing quality and optical properties suited to spectroscopy. No light spill was detectable from the holder and only minimal loss seen through the margin (edge) of the cuvette, which was a consistent loss due to the high manufacturing quality of the cuvette unit.**

Figures 3 & 4 and Lines 195-197: Please clarify what is meant by “…all MAI data… were regressed against Hbt…”. What statistical treatment is being done? Or are the data simply plotted together on the same graph? Also, please clarify which data is indicated by the markers, solid line, and dashed lines. It seems that the authors are plotting (Hbt vs. NaNO2) data with (MetHb vs. NaNO2) data, but NaNO2 concentrations are not shown in the plot.

This follows some partial explanation provided above:

**Regression of our MAI with Hbt (using the CeraChek instrument (sensitive only to HbFe2+) was an independent method to demonstrate that the MIA generated *in vivo* was indeed strongly and linearly correlated with declines in HbFe2+, right up until a lethal outcome was observed. These are the data we refer to.**

**We have attempted to reduce confusion here by making some further alterations in the MS relating to what we call Hbt (as denoted by the CeraChek) with what is more correctly expressed as HbFe2+. Although the CeraChek device seeks total Hb (Hbt), it is very useful as it *does not measure pre-existing MetHb (HbFe3+) in the sample*. We selected this approach because the device had the unique ability to produce regression data on declining HbFe2+ as MetHb (HbFe3+) increased. This is somewhat unconventional, yet we needed a way to independently validate/calibrate small (3 micro L) samples. No standard instrumentation (e.g. oximeters) existed to allow this at the pint of sample collection. The smallest oximeter sample volume we found (50 micro L sample) was the Avoximeter unit that would not routinely generate data in bird blood – mostly it generated error readings. It was not possible to validate against other devices with much larger blood volumes. Hence, our approach also needed to find a way to validate data in extremely small samples. The CeraChek biosensor allowed that to be done with no more than 1 micro L blood samples.**

**We have now changed the Y-axis title to refer to “HbFe^2+^”**

**We stipulate now that regression is with 95% confidence:**

**“Regression of absorbance data (MAI Eq. 1. = 575 nm – 630 nm) with total haemoglobin (HbFe^2+^) at T1 (60 min) and T2 (180 min) for all birds dosed with PAPP (⚫) at T0 with ST_99_ (0.66), LT_50_ (0.53) and T0 mean with 95% confidence. Table 3 contains corresponding statistical information (a. Chicken; b. Japanese quail; c. Duck; d. Pukeko; e. Black-Backed Gull; f. Takahē; g. Eastern Rosella; h. Weka and i. Kiwi).**

**In the discussion we now say:**

**“Requiring only a 1 µL blood sample, the Cera-Chek biosensor provided a useful relative indicator of declining Hb(Fe^2+^) in birds dosed with PAPP that closely corresponded with the declining MAI values enabling a routine two-factor validation of data taken from all bird species.”**

Figure 5: Again, a nonzero baseline for the HbO spectrum (figure 5a) this time in freshly drawn chicken blood indicates scattering due to sample or sample holder. A change in OD at 575nm could be due to a reduction in scattering from cell lysis or sedimentation. This would complicate the interpretation of results reported as a change in HbO percentage with time and treatment. Additionally, the absence of tick labels for figure 5b makes it impossible to determine whether scattering is occurring in the sample.

**Thanks for pointing this out – yes, it seems a bit odd. Again, the samples had been light/dark calibrated and the standard settings and calibrations were done in each case. We looked back at the data and settings in case we had omitted to remove an off-set or made an error in the graphical display. The explanation for this seems to be that in the 24 hour old sample the baseline in the infra-red also collapsed i.e. this is a segment of a far larger spectra that has zero baselines that is not shown. We toyed with re-interpreting these data and re-setting the baseline. Although we did not find obvious haemolysis, we don’t really know what changes have happened other than an increase in deoxyHb and a loss of HbO. Several other changes (Heinz bodies? Sedimentation? Coagulation?) could explain this, but we don’t have any other data to support this beyond speculation. One of the aspects is the shallow HbO peaks in the T0 sample – which commonly happens with birds caged in the lab after anaesthesia.**

**We’d rather leave it as is, but have noted in the results:**

**“Overall, large changes to the absorbance values over the entire spectra were observed in day-old blood, although visual and spectroscopic examination found no obvious haemolysis present (Fig 5).”**

**In the discussion we now state:**

**“Unlike mammalian blood, substantial *in vitro* sample degradation in chicken blood was observed within 24 hours, denoted by the loss of HbO absorbance peaks and increases in deoxyHb compared to freshly drawn samples. This is consistent with observations that avian erthyrocytes consume oxygen some 7-10 times faster than mammalian blood (****34****). Although no hemolysis was detected in our day-old blood sample, widespread changes to the absorbance values over the full spectra were observed, along with frequent error messages from the CO-oximeter when day-old blood was used. Chicken blood may be subject to rapid coagulation and is difficult to store even for short periods (****35****). Anticoagulants used for the storage of nucleated erythrocytes are also associated with changes in blood values and hemolysis (****36****).”**

**Both Fig 5a and 5b have been re-drawn.**

6. PLOS authors have the option to publish the peer review history of their article ([what does this mean?](https://journals.plos.org/plosone/s/editorial-and-peer-review-process#loc-peer-review-history)). If published, this will include your full peer review and any attached files.


If you choose “no”, your identity will remain anonymous but your review may still be made public.


**Do you want your identity to be public for this peer review?** For information about this choice, including consent withdrawal, please see our [Privacy Policy](https://www.plos.org/privacy-policy).

Reviewer #1: **Yes:** Stuart R McLean.

Reviewer #2: No

Reviewer #3: No
